# Comparative metabolic profiling of wild *Cordyceps* species and their substituents by liquid chromatography-tandem mass spectrometry

**DOI:** 10.3389/fphar.2022.1036589

**Published:** 2022-11-24

**Authors:** Shan Guo, Manting Lin, Di Xie, Wenqing Zhang, Mi Zhang, Li Zhou, Sheng Li, Hankun Hu

**Affiliations:** ^1^ Department of Biological Repositories, Zhongnan Hospital of Wuhan University, Wuhan, Hubei, China; ^2^ Department of Pharmacy, Zhongnan Hospital of Wuhan University, Wuhan, Hubei, China; ^3^ Department of Pharmacy, Xiamen Maluan Bay Hospital, Xiamen, Fujian, China; ^4^ Animal Biosafety Level III Laboratory, Wuhan University School of Basic Medical Sciences, Wuhan, Hubei, China; ^5^ Department of Urology, Zhongnan Hospital of Wuhan University, Wuhan, Hubei, China; ^6^ Tumor Precision Diagnosis and Treatment Technology and Translational Medicine, Hubei Engineering Research Center, Wuhan, Hubei, China; ^7^ School of Pharmaceutical Sciences, Wuhan University, Wuhan, Hubei, China

**Keywords:** wild *Cordyceps* species, cultivated *Cordyceps* species, mycelia, metabolic profiling, liquid chromatography-tandem mass spectrometry

## Abstract

*Cordyceps* is a genus of ascomycete fungi and used widely in fungal drugs. However, in-depth studies of the metabolites of wild *Cordyceps* species and their substituents are lacking. In this study, a liquid chromatography-tandem mass spectrometry (LC-MS/MS)-based metabolomics analysis was carried out to comprehensively profile the metabolites in wild Chinese *Cordyceps* species (*Ophiocordyceps sinensis* (Berk.) G.H. Sung, J.M. Sung, Hywel-Jones and Spatafora 2007) from Naqu (NCs) and Yushu (YCs) and their substituents including artificially cultivated *Cordyceps* species (CCs) and mycelia. A total of 901 metabolites were identified in these samples, including lipids, amino acids, nucleosides, carbohydrates, organic acids, coenzymes, vitamins, alkaloids and their derivatives. Univariate and multivariate statistical analyses revealed remarkable differences and significantly different metabolites among them. Seventy amino acid-relevant metabolites were analyzed quantitatively in four samples for the first time. The four samples contained abundant L-glutamic acid and oxidized glutathione as well as multiple unique amino acid-relevant metabolites (e.g., 3-chloro-L-tyrosine, 6-aminocaproic acid, L-theanine, anserine, γ-glutamyl-cysteine). Collectively, our study provides rich metabolic information of wild *Cordyceps* species and their substituents, which could facilitate their quality control and optimal utilization.

## Introduction

*Cordyceps* is a genus of ascomycete fungi. *Cordyceps* species are highly valued tonic foods and well-known fungal drugs. Studies have demonstrated the diverse pharmaceutical effects of *Cordyceps* species-based products (Kuo et al., 1996; Zhang et al., 2005; Hsu et al., 2008; Yang et al., 2011; Zhang et al., 2014; Mi et al., 2018; Cheng et al., 2020) and revealed various chemical constituents, such as nucleosides, lipids, saccharides, mannitol, and amino acids (Zhao et al., 2014; Liu et al., 2015).

However, *Cordyceps* species are rarely found in the wild, and their substituents are desired (Li et al., 2019). The fermented mycelia of wild *Cordyceps* species are popular, and have been made into medical and functional products in the forms of capsules, tablets, or granulations (Dong et al., 2015; Zhao et al., 2020). Besides, Sunshine Lake Pharma (Dongguan, China) has undertaken artificial cultivation of Chinese *Cordyceps* species. *Ophiocordyceps sinensis* (Berk.) G.H. Sung, J.M. Sung, Hywel-Jones & Spatafora 2007 (known previously as *Cordyceps sinensis*) is a fungus that grows on insects in the family Ophiocordycipitaceae (Li et al., 2019). *O. sinensis* is a form of wild *Cordyceps* species. The annual yield of cultured *Cordyceps* species (CCs) reaches tens of tons, accounting for ≥20% of the total natural resource (Li et al., 2019). However, whether wild *Cordyceps* species (e.g., *O. sinensis*) and such substituents have chemical homogeneity is controversial.

Metabolomics analysis has become a powerful approach for large-scale study of metabolites in biological samples (Patti et al., 2012). In general, two analytical methods are used to conduct metabolomics analysis: nuclear magnetic resonance (NMR) spectroscopy and mass spectrometry (MS) (Perez et al., 2021). Compared with MS, NMR provides unique structural information. Nonetheless, it has limited sensitivity when measuring a large number of metabolites with a wide dynamic range (Lu et al., 2019; Perez et al., 2021). With regard to MS-based metabolic profiling, gas chromatography (GC) and liquid chromatography (LC) are hyphenated to MS for more accurate identification of metabolites. GC-MS often requires further derivatization of metabolites, and makes them more volatile and more thermally stable (Smart et al., 2010; Zhang et al., 2020). Nevertheless, LC-MS can detect thermally labile metabolites directly without derivatization, and is used more widely for metabolite profiling. LC-MS-based metabolomics analysis has been applied to analyze a certain class of metabolites (e.g., lipid, nucleoside, nucleobase, nucleotide, protein) (Fan et al., 2006; Mi et al., 2016; Cheng et al., 2017; Zhang et al., 2018; Jia et al., 2019; Lin et al., 2022) or dozens of metabolites (Wang et al., 2015; Zhang et al., 2015; Chen et al., 2018; He et al., 2019; Yao et al., 2019) in wild *Cordyceps* species and/or their substituents. However, few studies have profiled hundreds of metabolites in wild *Cordyceps* species or cultivated *Cordyceps* species and their mycelia for more comprehensive assessment of their chemical composition and alterations.

In this study, we leveraged liquid chromatography-electrospray ionization-tandem mass spectrometry (LC-ESI-MS/MS) to comprehensively survey metabolites in wild *Cordyceps* species (*O. sinensis*) from Naqu (Tibet, China; NCs) and Yushu (Qinghai, China; YCs), CCs (*O. sinensis*), and mycelia (*Hirsutella sinensis* X.J. Liu, Y.L. Guo, Y.X. Yu & W. Zeng 1989) from Bailing capsules (BLs). Using univariate and multivariate statistical analyses, chemical differences among NCs, YCs, CCs, and BLs were evaluated. Furthermore, differential metabolites among four samples were screened out and enrichment analysis using the Kyoto Encyclopedia of Genes and Genomes (KEGG) database was undertaken. Besides, 70 amino acid-relevant metabolites in these four samples were analyzed quantitatively. Taken together, this work provides a holistic insight into the metabolic differences in wild *Cordyceps* species and their substituents.

## Materials and methods

### Materials and reagents

Wild *Cordyceps* species (*O. sinensis* (Berk.) G.H. Sung, J.M. Sung, Hywel-Jones & Spatafora 2007), from Naqu (NCs) and Yushu (YCs) were acquired from Hubei Tianji Pharmaceuticals (Hubei, China) and Qinghai Yuanzu Biological Technology (Qinghai, China), respectively. Artificially cultivated *Cordyceps* species (*O. sinensis*; catalog number = 200111-I01) was purchased from Sunshine Lake Pharma (Guangdong, China). BLs containing *H. sinensis* X.J. Liu, Y.L. Guo, Y.X. Yu & W. Zeng 1989 (1911355) were obtained from Zhongmei East China Pharmaceuticals (Zhejiang, China). Methanol (I1069707004) and acetonitrile (JA095830) were sourced from Merck (Darmstadt, Germany). Formic acid (RH208519) was purchased from Aladdin (Shanghai, China). Standards for quantitative analysis of amino acid-relevant metabolites are shown in Supplementary Table S1. [2H2]L-threonine [IsoReag (Shanghai, China); 21J161-G1] was used as an internal standard and added to calibrate as-prepared *Cordyceps* samples, compensating for losses during sample preparation or variability during analytical determination.

### Liquid chromatography-tandem mass spectrometry for metabolic profiling of wild and cultivated species of *Cordyceps* and mycelia

To extract metabolites, 50 mg of each sample was first weighed and homogenized with steel balls at 30 Hz for 3 min. Then, 1 ml of a methanol:water mixture (7:3) was added, and the mixture was vortex-mixed for 5 min followed by centrifugation (12,000 rpm, 10 min, 4°C). Subsequently, the supernatant (400 μl) was collected and stored at −20°C overnight. Finally, the supernatant was centrifuged again (12,000 rpm, 3 min, 4°C), and 200 μl of supernatant pipetted for LC-MS.

Chromatographic separation was undertaken on a Shim-pack CBM30A ultra-high-pressure liquid chromatography (UPLC) system (Shimadzu, Kyoto, Japan). An Acquity HSS T3 C18 column (2.1 mm × 100 mm, 1.8 μm; Waters, Milford, MA, United States) was used for LC and its temperature were maintained at 40°C. Mobile phase A was H_2_O with 0.1% formic acid. Mobile phase B was acetonitrile with 0.1% formic acid. Gradient elution was: 0–10 min, 5%–90% B; 10–11 min, 90% B; 11–11.1 min, 90%–5% B; 11.1–14 min, 5% B. The flow rate was 0.35 ml/min and the injection volume was 2 μl.

A Triple Quad^™^ 6500+ MS (Sciex, Framingham, MA, United States) instrument was operated in electrospray ionization in positive (ESI+) and negative (ESI−) modes with the following parameters: positive ion spray voltage, 5500 V; negative ion spray voltage, −4500 V; source temperature, 500°C; ion source gas 1, 50 psi; ion source gas 2, 50 psi; curtain gas, 25 psi; collision gas, high. A specific set of multiple reaction monitoring (MRM) transitions was recorded for each period according to the metabolites eluted within this period.

### Liquid chromatography-tandem mass spectrometry for quantifying amino acid-relevant metabolites

Similarly, 50 mg of each sample was weighed and homogenized with steel balls at 30 Hz for 3 min. Then, 500 µl of a methanol:water (7:3) mixture containing [2H2]L-threonine was added. The mixture was vortex-mixed for 5 min followed by centrifugation (12,000 rpm, 10 min, 4°C). Subsequently, the supernatant (300 μl) was collected and stored at −20°C for 30 min. Finally, the supernatant was centrifuged again (12,000 rpm, 10 min, 4°C) and 200 μl of the supernatant was pipetted for LC-MS.

Chromatographic separation was achieved on an ExionLC AD UPLC system (Sciex) with an Acquity BEH Amide column (2.1 mm × 100 mm, 1.7 μm). The column temperature was maintained at 40°C. Mobile phase A was H_2_O with ammonium acetate (2 mM) and 0.04% formic acid. Mobile phase B was acetonitrile with ammonium acetate (2 mM) and 0.04% formic acid. Gradient elution was: 0–1.2 min, 90% B; 1.2–9 min, 90%–60% B; 9–10 min, 60%–40% B; 10–11 min, 40% B; 11–15 min, 40%–90% B. The flow rate was 0.35 ml/min, and the injection volume was 2 μl.

Acquisition of MS data was undertaken on a Triple Quad^™^ 6500+ MS system (Sciex) in MRM mode with the following parameters: positive ion spray voltage, 5500 V; negative ion spray voltage, −4500 V; source temperature, 550°C; ion source gas 1, 50 psi; ion source gas 2, 50 psi; curtain gas, 35 psi; collision gas, medium.

### Data processing

Metabolites were identified by matching the retention time, precursor ions/product ions, and MS/MS spectra to the self-compiled MetWare database (MetWare Biological Science and Technology, Wuhan, China) and public databases [METLIN (https://massconsortium.com/), Human Metabolome Database (HMDB; https://hmdb.ca/), KEGG (www.genome.jp/)] using Analyst 1.6.3 (Sciex). The integration and calibration of chromatographic peaks were done with MultiQuant 3.0.3 (Sciex).

### Statistical analyses

Principal component analysis (PCA) and orthogonal partial least squares-discriminant analysis (OPLS-DA) were conducted in R (R Institute for Statistical Computing, Vienna, Austria) using the statistics function “prcomp” and “MetaboAnalystR” package, respectively. Hierarchical clustering analysis (HCA) and the Pearson correlation coefficient were carried out by the R package “ComplexHeatmap.”

## Results and discussion

### Liquid chromatography-tandem mass spectrometry-based wide-targeted metabolomics analysis for the characterization of metabolites in wild *Cordyceps* species and their substituents

LC-MS-based strategies for metabolomics analysis can be targeted or untargeted (Patti et al., 2012). Untargeted metabolomics analysis is dependent upon high-resolution MS to measure metabolites without bias. Typically, targeted metabolomics analysis uses MRM on MS/MS to analyze *a priori* defined metabolites with high sensitivity and quantitative reproducibility. In this work, a LC-MS/MS-based wide-targeted metabolomics analysis was employed to comprehensively identify metabolites and assess their alterations in wild Chinese *Cordyceps* species (*O. sinensis* (Berk.) G.H. Sung, J.M. Sung, Hywel-Jones & Spatafora 2007; NCs and YCs) and their substituents, including artificially cultivated *Cordyceps* species (CCs) and fermented mycelia (BLs). Total ion chromatographs of sample metabolomic analysis in ESI+ and ESI− modes are shown in Supplementary Figure S1. A total of 901 metabolites were detected in pooled quality control (QC) samples comprising extracts of NCs, YCs, CCs, and BLs. Table 1 shows that the chemical constituents of samples included lipids, amino acids, nucleotides, carbohydrates, organic acids, vitamins, and their derivatives or other small molecules. Notably, more lipids, amino acids, organic acids, and their derivatives were detected.

**TABLE 1:** Metabolites detected in pooled QC samples comprising extracts of NCs, YCs, CCs, and BLs.

Classes	Subclasses	Number	Total
Lipids	Glycerophospholipids	63	181
	Glycerolipids	10	
	Sphingolipids	1	
	Oxidized lipids	28	
	Fatty acyls	79	
Amino acid and its derivatives	Amino acids	36	210
	Amino acid derivatives	92	
	Small peptides	82	
Nucleotide and its derivatives	Nucleotide and its derivatives	83	83
Carbohydrate and its derivatives	Phosphate sugars	13	51
	Sugar alcohols	7	
	Sugars	22	
	Sugar acids	6	
	Sugar derivatives	3	
Organic acid and its derivatives	Sulfonic acids	6	135
	Phosphoric acids	7	
	Organic acid and its derivatives	122	
Coenzyme and vitamins	Coenzyme and vitamins	23	23
Alkaloids	Alkaloids	1	1
Others	Benzene and substituted derivatives	69	217
	Alcohols and amines	54	
	Heterocyclic compounds	49	
	Aldehyde, ketones, and esters	24	
	Tryptamines, cholines, and pigments	6	
	Hormones and hormone related compounds	4	
	Others	11	

**FIGURE 1:**
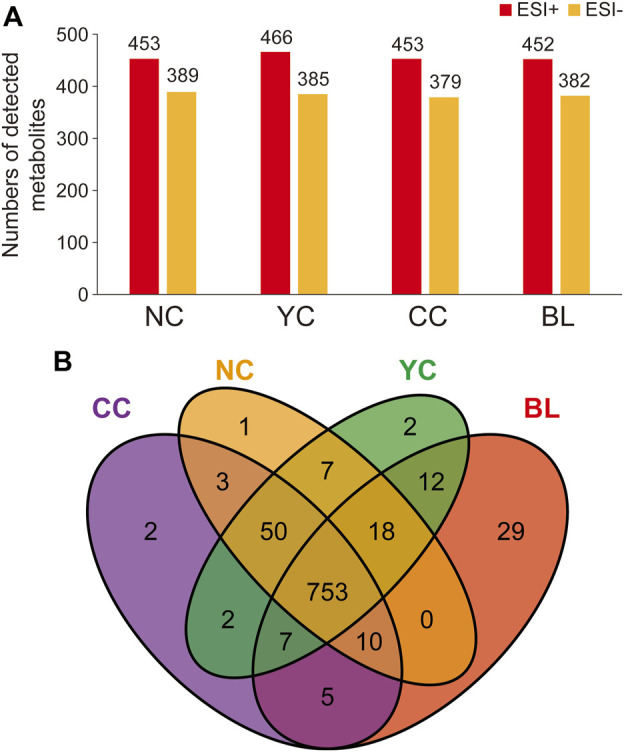
Metabolites identified in NCs, YCs, CCs, and BLs. **(A)** Numbers of metabolites detected from NCs, YCs, CCs, and BLs in ESI+ and ESI- modes, respectively. **(B)** Venn diagram of metabolites detected from NCs, YCs, CCs, and BLs.

Specifically, in ESI + mode, 453, 466, 453, and 452 metabolites were detected in NCs, YCs, CCs, and BLs, respectively. In ESI− mode, 389, 385, 379, and 382 metabolites were detected in NCs, YCs, CCs, and BLs, respectively (Figure 1). Detailed information of metabolites detected in NCs, YCs, CCs, and BLs is displayed in Supplementary Table S2. A Venn diagram highlighted that 753 out of 901 metabolites were detected in these four samples (Figure 1B). Importantly, some widely used bioactive constituents or biomarkers were identified in all samples, such as nucleosides (e.g., cordycepin, adenosine), ergosterol, D-mannitol, and amino acids (e.g., tryptophan, glutamic acid) (Li et al., 2006; Yue et al., 2013; Zhao et al., 2014; Liu et al., 2015). Besides, 1, 2, 2, and 29 metabolites were only detected in NCs, YCs, CCs, and BLs, respectively (Figure 1B; Table 2). Collectively, we detected a wide variety of metabolites with large amounts in wild and cultivated *Cordyceps* species and mycelia, which facilitated comprehensive assessment of their differences.

**TABLE 2:** Metabolites found unique to NCs, YCs, CCs, and BLs, respectively.

Cordyceps samples	Unique metabolites	Subclasses
NC	3-Ketosphingosine	Alcohols and amines
YC	18-Hydroxyoleate	Fatty acyls
	N′-Formylkynurenine	Amino acid derivatives
CC	Carnitine C5:DC	Fatty acyls
	α-Cyclopiazonic acid	Benzene and substituted derivatives
BL	1-O-Palmitoyl-2-O-acetyl-sn-glycerol-3-phosphate choline	Glycerophospholipids
	1-(1Z-Hexadecenyl)-sn-glycero-3-phosphocholine	Glycerophospholipids
	LPA (18:2)	Glycerophospholipids
	(±)16-HETE	Oxidized lipids
	20-HETE	Oxidized lipids
	Xanthurenic acid	Amino acid derivatives
	S-(Indolylmethylthiohydroximoyl)-L-cysteine	Amino acid derivatives
	γ-Glutamyl-L-putrescine	Amino acid derivatives
	Histidyl-threoninyl-lysine	Small peptides
	Threoninyl-tyrosyl-lysine	Small peptides
	Glutaminyl-valine	Small peptides
	L-Alanyl-L-valine	Small peptides
	2-Amino-4,6-pteridinediol	Nucleotide and its derivatives
	Creatinine	Organic acid and its derivatives
	Indole-3-pyruvic acid	Organic acid and its derivatives
	3-Methoxyphenylacetic acid	Organic acid and its derivatives
	5-Methyltetrahydrofolate	Organic acid and its derivatives
	Methyl nicotinate	Coenzyme and vitamins
	4-Acetamidobutanoyl-CoA	Coenzyme and vitamins
	Acetaminophen glucuronide	Alcohols and amines
	5,7,22,24 (28)-Ergostatetraenol	Alcohols and amines
	1,3-Diphenylguanidine	Alcohols and amines
	N-Butylaniline	Alcohols and amines
	Isoxanthopterin	Heterocyclic compounds
	3-Indolepropionic acid	Heterocyclic compounds
	1-Methyl-hydantoin	Heterocyclic compounds
	α-[3-[(Hydroxymethyl)nitrosoamino]propyl]-3-pyridinemethanol	Heterocyclic compounds
	Methyl propyl disulfide	Others
	8,8a-Deoxy oleane	Others

### Multivariate statistical analyses for assessment of differences between wild *Cordyceps* species and their substituents

**FIGURE 2:**
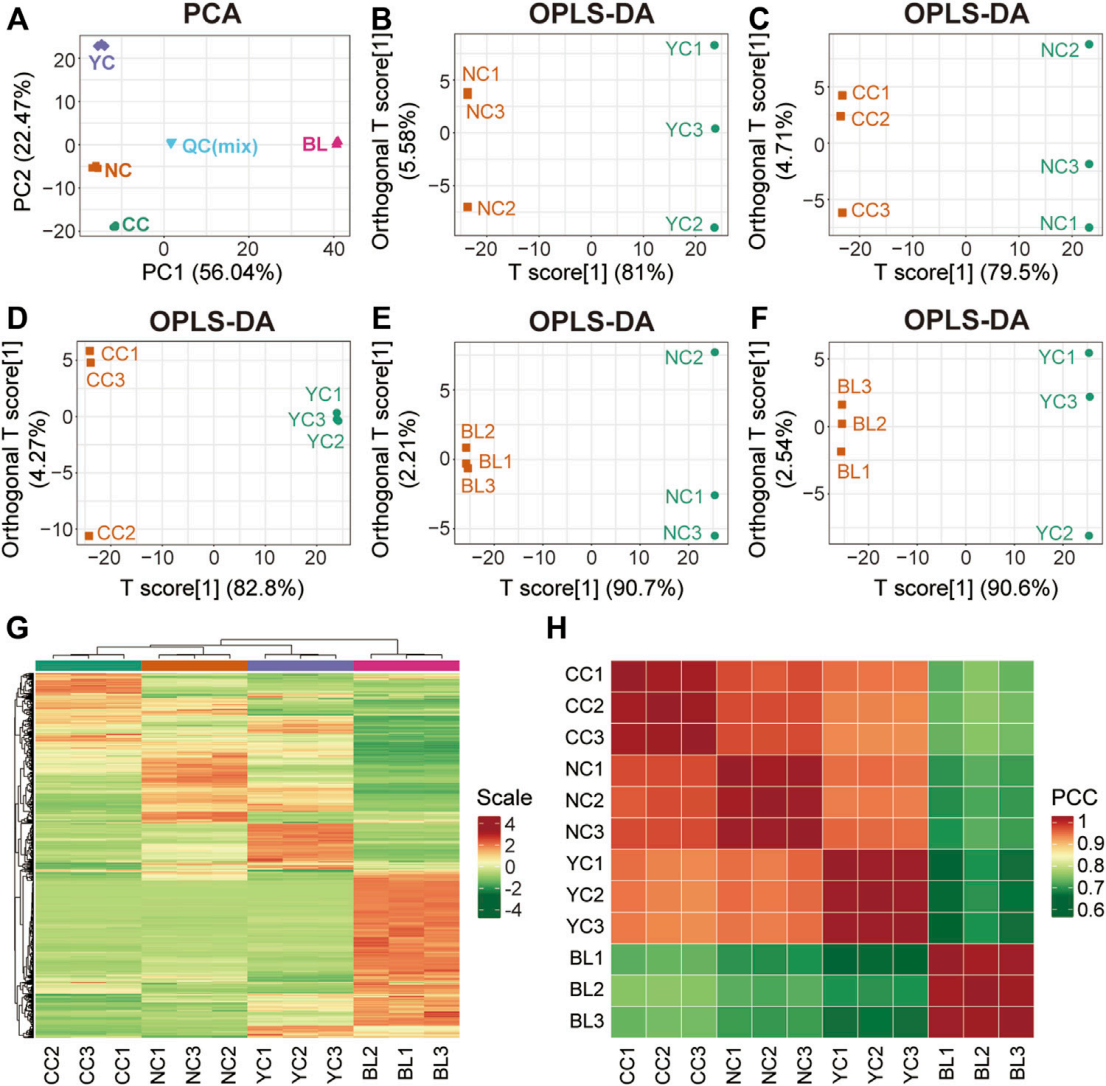
Multivariate statistical analyses of metabolites detected in NCs, YCs, CCs, and BLs. **(A)** PCA result. **(B–F)** Plots showing OPLS-DA scores. **(G)** Heatmap of hierarchical clustering analysis. **(H)** Heatmap of correlation analysis. PCC, Pearson’s correlation coefficient.

To characterize differences between wild *Cordyceps* species and their substituents comprehensively, multivariate statistical analyses (PCA, OPLS-DA, clustering analysis, correlation analysis) were employed. Metabolites measured in QC samples with relative standard deviation <30% were used for subsequent analyses (Supplementary Table S2). Results of PCA (Figure 2A) and OPLS-DA (Figures 2B–F) clearly showed distinct sample clusters corresponding to NCs, YCs, CCs, and BLs, respectively. Besides, clustering analyses (Figure 2G) and correlation analyses (Figure 2H) indicated differential metabolites among NCs, YCs, CCs, and BLs. Notably, the differences between CCs and wild *Cordyceps* species (NCs and YCs) were less than those between BLs and wild *Cordyceps* species. The similarity between CCs and NCs was higher than that between CCs and YCs (Figure 2H). Taken together, these results suggested that metabolic differences were present among NCs, YCs, CCs, and BLs.

### Differential metabolites between *Cordyceps* from Naqu and Yushu

**FIGURE 3:**
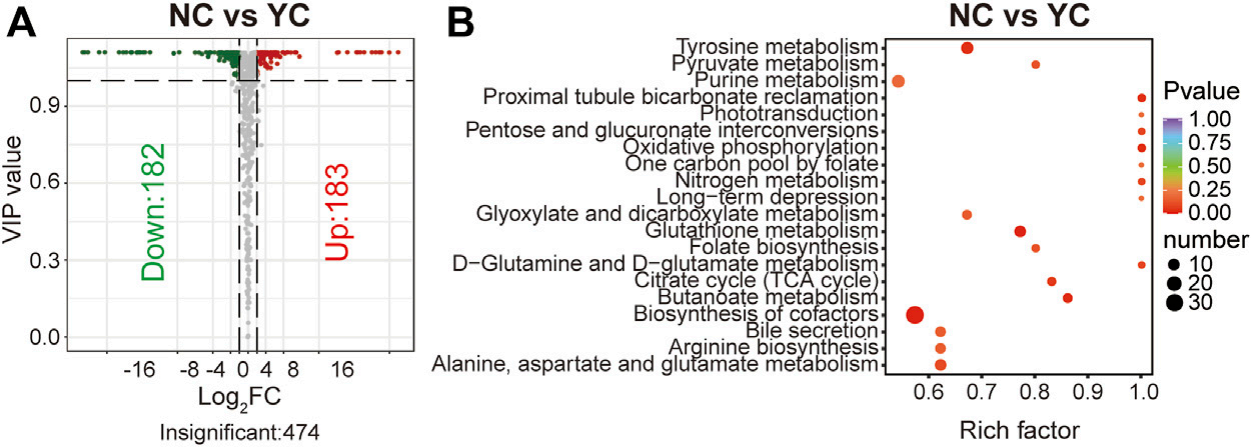
Differential analyses between NCs and YCs. **(A)** Volcano plot of differential metabolites between NCs and YCs. Differential metabolites were screened based on VIP ≥1 and FC ≥ 2 or ≤0.5. The fold change was the ratio of NCs to YCs. **(B)** Enrichment analysis of differential metabolites between NCs and YCs using the KEGG database. The top-20 enriched metabolic pathways (with *p*-values) are displayed.

Molecular features with variable importance in projection (VIP) ≥1 and fold change (FC) ≥2 or ≤0.5 were defined as significantly differential metabolites between two samples of *Cordyceps* species. A total of 365 differential metabolites (183 were up-regulated and 182 were down-regulated) between NCs and YCs were documented (Figure 3A). Furthermore, except for one alkaloid, all detected metabolite classes had an upward or downward trend in level (Supplementary Table S3). Furthermore, enrichment analysis using the KEGG database displayed that more differential metabolites between NCs and YCs were associated with biosynthesis of cofactors (Figure 3B). However, nine glycerolipids (MG (18:2 (9Z, 12Z)/0:0/0:0) [rac], glycidyl oleate, MAG (16:1) isomer, MAG (16:1), MG (20:5) isomer, MG (18:1 (9Z)/0:0/0:0) [rac], glycine linoleate, (±) 1,2-sebacyl glycerol (10:0), 2-palmitoyl-rac-glycerol) and one sphingolipid (Sphinganine 1-phosphate) were detected in both types of wild *Cordyceps* species. Besides, the contents of bioactive cordycepin (i.e., 3′-deoxyadenosine) and D-mannitol (i.e., cordycepic acid) were higher in NCs than YCs. Collectively, these data suggested that wild *Cordyceps* species from different habitats had some differential metabolites, indicating that local unique ecological factors may affect the chemical constituents of wild *Cordyceps* species.

### Differential metabolites between wild and cultivated species of *Cordyceps*

**FIGURE 4:**
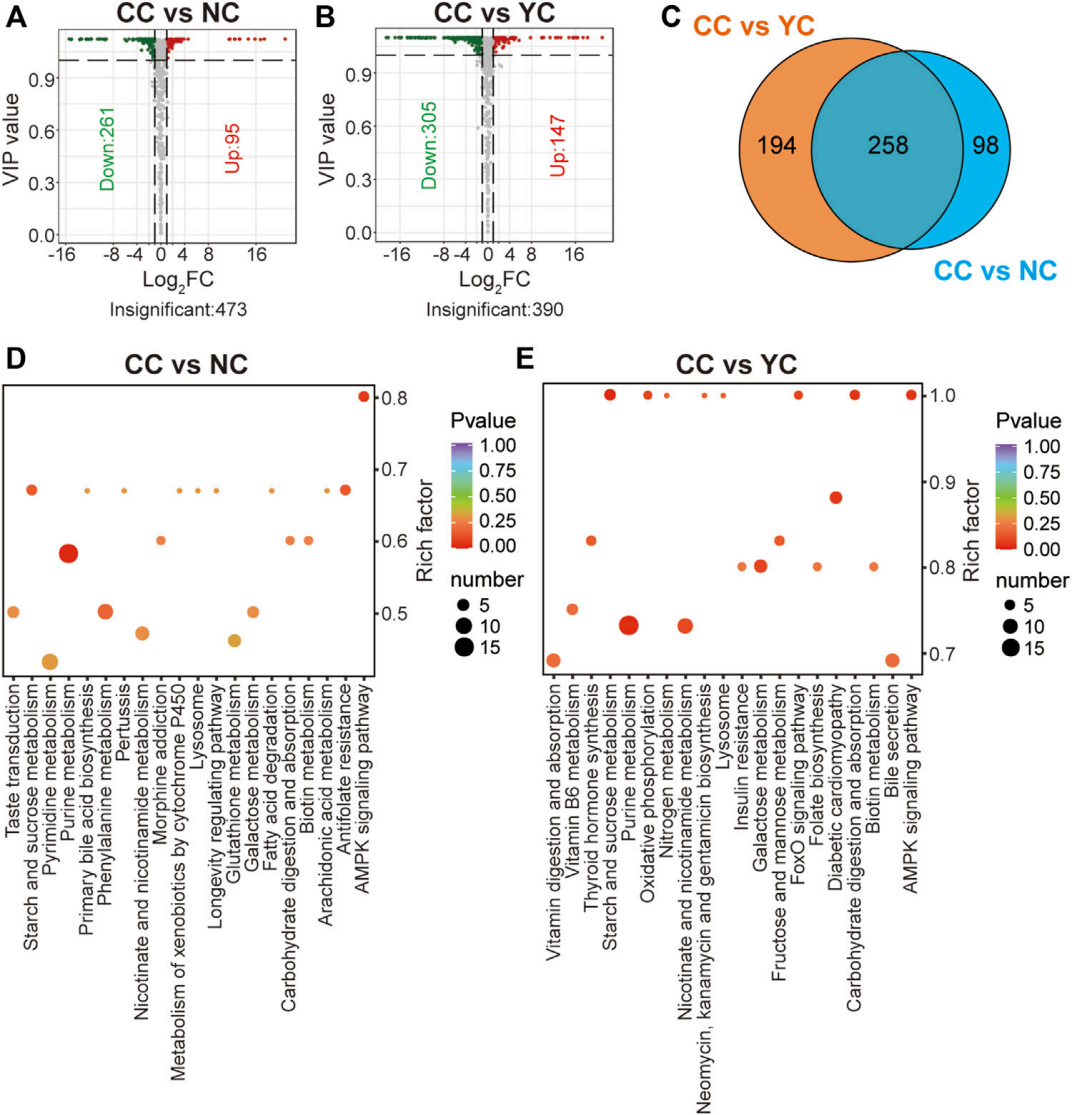
Differential analyses between CCs and wild *Cordyceps* species. **(A)** Volcano plot of differential metabolites between CCs and NCs. Differential metabolites were screened based on VIP ≥1 and FC ≥ 2 or ≤0.5. The fold change was the ratio of CCs to NCs. **(B)** Volcano plot of differential metabolites between CCs and YCs. Differential metabolites were screened based on VIP ≥1 and FC ≥ 2 or ≤0.5. The fold change was the ratio of CCs to YCs. **(C)** Venn diagram of differential metabolites between CCs and wild *Cordyceps* species. **(D)** Enrichment analysis of differential metabolites between CCs and NCs using the KEGG database. The top-20 KEGG enriched metabolic pathways (with *p*-values) are displayed. **(E)** Enrichment analysis of differential metabolites between CCs and YCs using the KEGG database. The top-20 KEGG enriched metabolic pathways (with *p*-values) are displayed.

Similarly, 356 significant differential metabolites (95 were up-regulated and 261 were down-regulated) were found between CCs and NCs, and 452 remarkably altered metabolites (147 were up-regulated and 305 were down-regulated) were found between CCs and YCs (VIP ≥1 and FC ≥ 2 or ≤0.5) (Figures 4A,B). Among them, there were 258 overlapped differential metabolites (including lipids, amino acids, nucleotides) between CCs and wild *Cordyceps* species (Figure 4C) and they are emboldened in Supplementary Tables S4, S5. Of note, the content of ergosterol was lower in CCs than in both types of wild *Cordyceps* species. Besides, six sugar acids (D-glucoronic acid, L-gulonolactone, D-glucarate, L-arabinonic acid-1,4-lactone, mucic acid, gluconic acid), and two sugar derivatives (N-acetyl-D-glucosamine, methyl beta-D-galactopyranoside) detected in wild and cultivated species of *Cordyceps* displayed no differences. Moreover, enrichment analyses using the KEGG database clearly showed that more significant differential metabolites between CCs and wild *Cordyceps* species were associated mainly with purine metabolism (Figures 4D,E). These results showed that CCs and wild *Cordyceps* species had different chemical constituents. On the one hand, this finding will provide promising indicators to discriminate artificially cultivated *Cordyceps* species from wild *Cordyceps* species (Zhang et al., 2015). On the other hand, this finding may encourage study of whether these differential metabolites between CCs and wild *Cordyceps* species are relevant to differences in their pharmacology.

### Differential metabolites between bailing capsules and wild *Cordyceps* species

**FIGURE 5:**
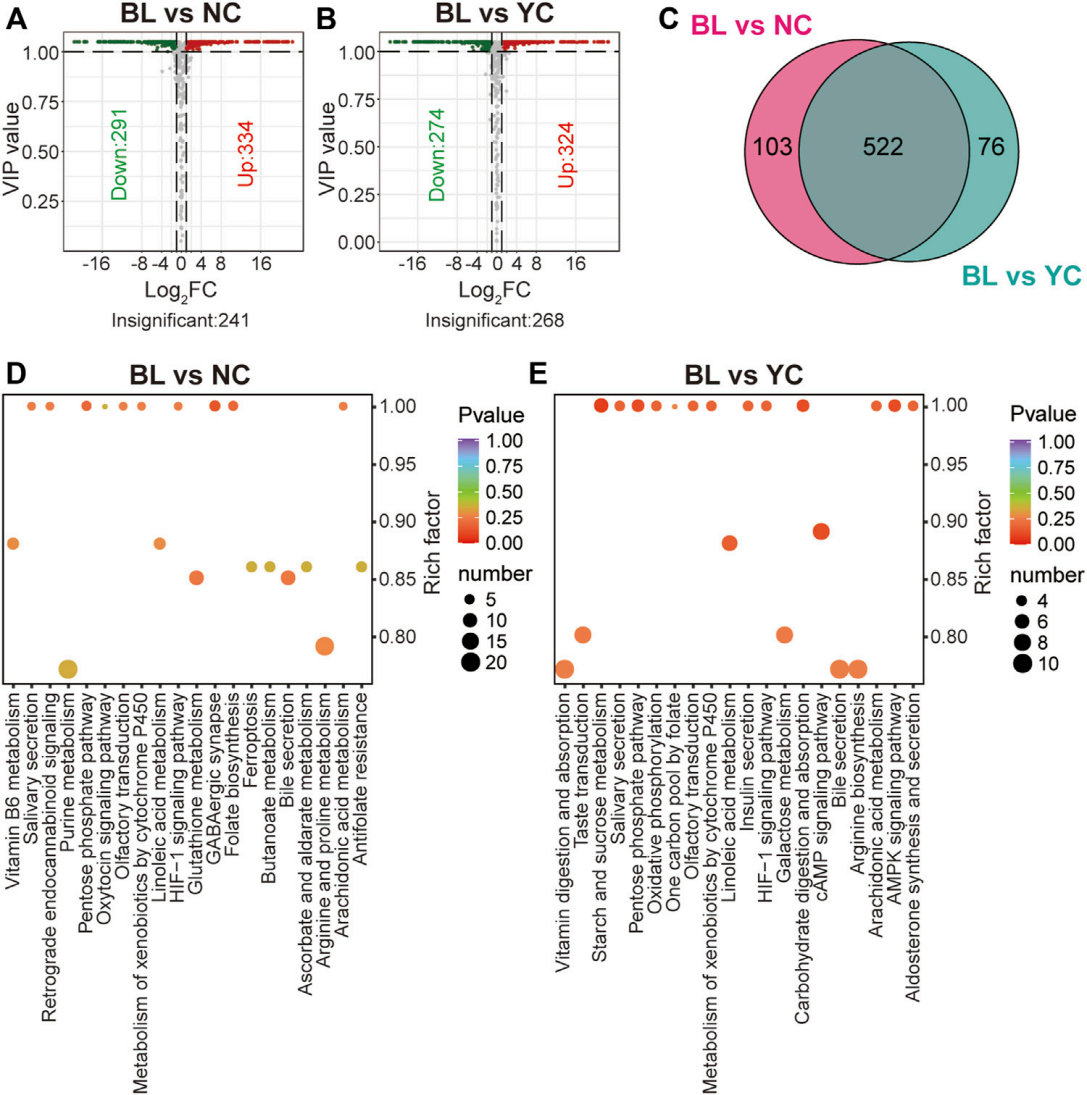
Differential analyses between BLs and wild *Cordyceps* species **(A)** Volcano plot of differential metabolites between BLs and NCs. Differential metabolites were screened based on VIP ≥1 and FC ≥ 2 or ≤0.5. The fold change was the ratio of BLs to NCs. **(B)** Volcano plot of differential metabolites between BLs and YCs. Differential metabolites were screened based on VIP ≥1 and FC ≥ 2 or ≤0.5. The fold change was the ratio of BLs to NCs. **(C)** Venn diagram of differential metabolites between BLs and wild *Cordyceps* species. **(D)** Enrichment analysis of differential metabolites between BLs and NCs using the KEGG database. The top-20 enriched pathways (with *p*-values) are displayed. **(E)** Enrichment analysis of differential metabolites between BLs and YCs using the KEGG database. The top-20 enriched pathways (with *p*-values) are displayed.

Compared with NCs, 625 metabolites were altered significantly in BLs (VIP ≥1; FC ≥ 2 or ≤0.5; 334 were up-regulated; 291 were down-regulated) (Figure 5A; Supplementary Table S6). Besides, 598 significantly different metabolites (324 were up-regulated and 274 were down-regulated) were found between BLs and YCs (Figure 5B; Supplementary Table S7). Figure 5C shows 522 overlapped differential metabolites between BLs and wild *Cordyceps* species, and they are emboldened in Supplementary Tables S6, S7. There were more differential metabolites (including lipids, amino acids, nucleotides) between BLs and wild *Cordyceps* species (Figures 5A,B) than those between CCs and wild *Cordyceps* species (Figures 4A,B). For the well-known bioactive constituents, the cordycepin content was higher in BLs than in wild *Cordyceps* species, whereas the ergosterol content was lower in BLs. Likewise, these differential metabolites between BLs and wild *Cordyceps* species could facilitate discrimination of adulteration in the processed products of wild *Cordyceps* species (Zhang et al., 2020). Besides, enrichment analyses using the KEGG database showed that differential metabolites between BLs and NCs and between BLs and YCs were located to different pathways (Figures 5D,E), which indicated that wild *Cordyceps* species from different habitats had metabolomic differences.

### Quantitative analyses of amino acid-relevant metabolites in wild *Cordyceps* species and their substituents

**FIGURE 6:**
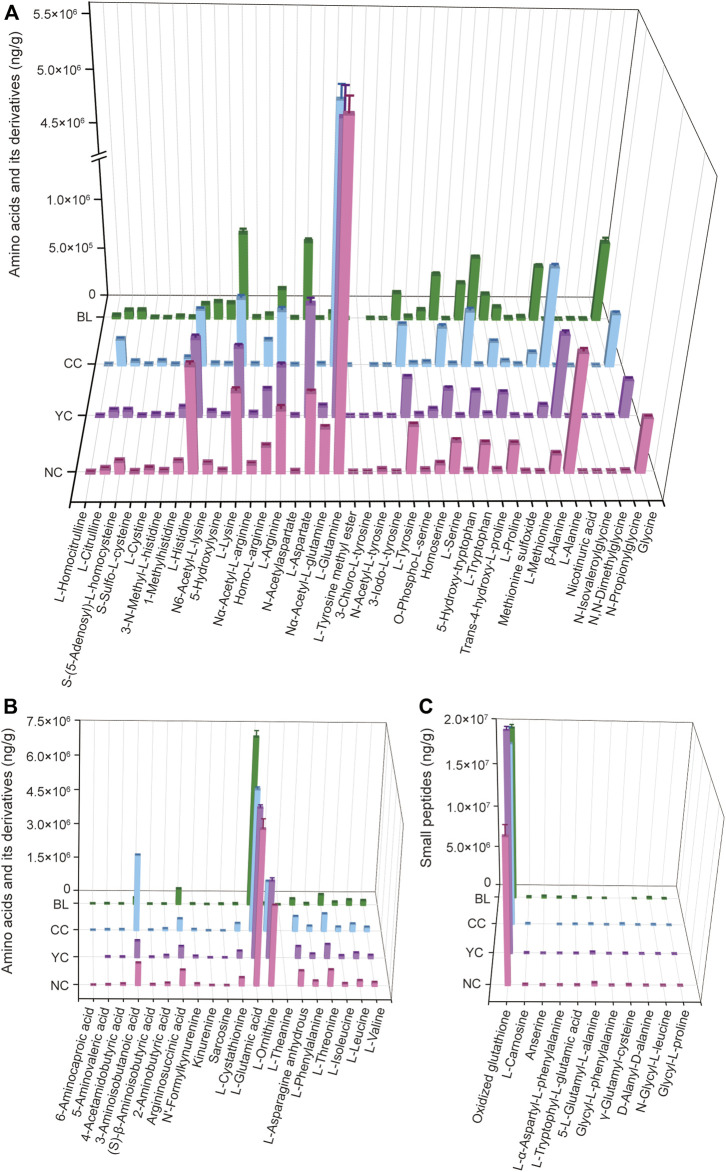
Absolute quantitative results of some amino acids and their derivatives **(A,B)** and small peptides **(C)** in wild and cultivated *Cordyceps* species and BLs.

Amino acids are important chemical and bioactive constituents in *Cordyceps* species (Zhang et al., 1991; Liu et al., 2015; Zhang et al., 2022) but have not been characterized comprehensively previously. Here, 70 amino acids and their relevant metabolites in four samples were analyzed quantitatively. Figures 6A,B show the quantitative results of some amino acids and their derivatives. Figure 6C displays the quantitative results of 11 small peptides. Compared with widely targeted metabolic analyses (Supplementary Figure S2), most of these amino acid-relevant metabolites detected by targeted quantitative analyses showed consistent trends in alteration across four samples (Figure 6). However, 11 amino acids and their derivatives were not detected by widely targeted metabolic analysis (nicotinuric acid, 5-hydroxy-tryptophan, homoserine, 3-iodo-L-tyrosine, 3-chloro-L-tyrosine, N-acetylaspartate, L-leucine, L-theanine, argininosuccinic acid, 2-aminobutyric acid, 4-acetamidobutyric acid) (Supplementary Figures S2A,B). Targeted quantitative analyses showed such undetected metabolites with relatively low abundance (Figures 6A,B).

Additionally, absolute quantitative results showed that L-glutamic acid and oxidized glutathione had a high level in wild and cultivated species of *Cordyceps* and mycelia (Figures 6B,C). Notably, the content of L-glutamine and L-ornithine was significantly higher in wild and cultivated species of *Cordyceps* than that in BLs (Figure 6A). Furthermore, some unique amino acid-relevant metabolites were noted: 1) 3-chloro-L-tyrosine was not detected in CCs and BLs, but was present in wild *Cordyceps* species (Figure 6A); 2) 6-aminocaproic acid was not detected in YCs but was present in NCs, CCs, and BLs (Figure 6B); 3) L-theanine was not detected in wild and cultivated species of *Cordyceps* but was present in BLs (Figure 6B); 4) anserine and γ-glutamyl-cysteine were not detected in CCs and BLs, respectively (Figure 6C).

## Conclusion

In summary, this work performed a comparative metabolic profiling to comprehensively characterize metabolites and assess their alterations in wild *Cordyceps* species (*O. sinensis* (Berk.) G.H. Sung, J.M. Sung, Hywel-Jones & Spatafora 2007) and their substituents. LC-MS/MS-based widely targeted approach measured 901 metabolites in *Cordyceps* samples, including lipids, amino acids, nucleosides, carbohydrates, organic acids, coenzymes, vitamins, alkaloids and their derivatives. Univariate and multivariate statistical analyses revealed metabolic differences among wild *Cordyceps* species from different habitats, cultivated *Cordyceps* species, and mycelia, and covered all the detected metabolite classes. Enrichment analyses using the KEGG database clearly showed differential metabolic pathways among four samples. Importantly, some amino acid-relevant metabolites were found to be unique to wild *Cordyceps* species (e.g., 3-chloro-L-tyrosine) or their substituents (e.g., L-theanine). These differences revealed among wild and cultivated *Cordyceps* species and mycelia could facilitate rational utilization and better QC.

## Data Availability

The original contributions presented in the study are included in the article/Supplementary Material, further inquiries can be directed to the corresponding authors.

## Supplementary material

SUPPLEMENTARY FIGURE S1 Total ion chromatograms of LC-MS/MS-based metabolic analysis of *Cordyceps* samples in positive and negative ion modes.

SUPPLEMENTARY FIGURE S2 Peak areas of amino acids and their derivatives **(A,B)** and small peptides **(C)** detected in NCs, YCs, CCs, and BLs.
