# Supplementary figures and images for: Comparative metabolic profiling of wild Cordyceps species and their substituents by liquid chromatography-tandem mass spectrometry
Source: Front Pharmacol. 2022 Nov 24;13:1036589. doi: 10.3389/fphar.2022.1036589 (PMC9729555; doi:10.3389/fphar.2022.1036589)

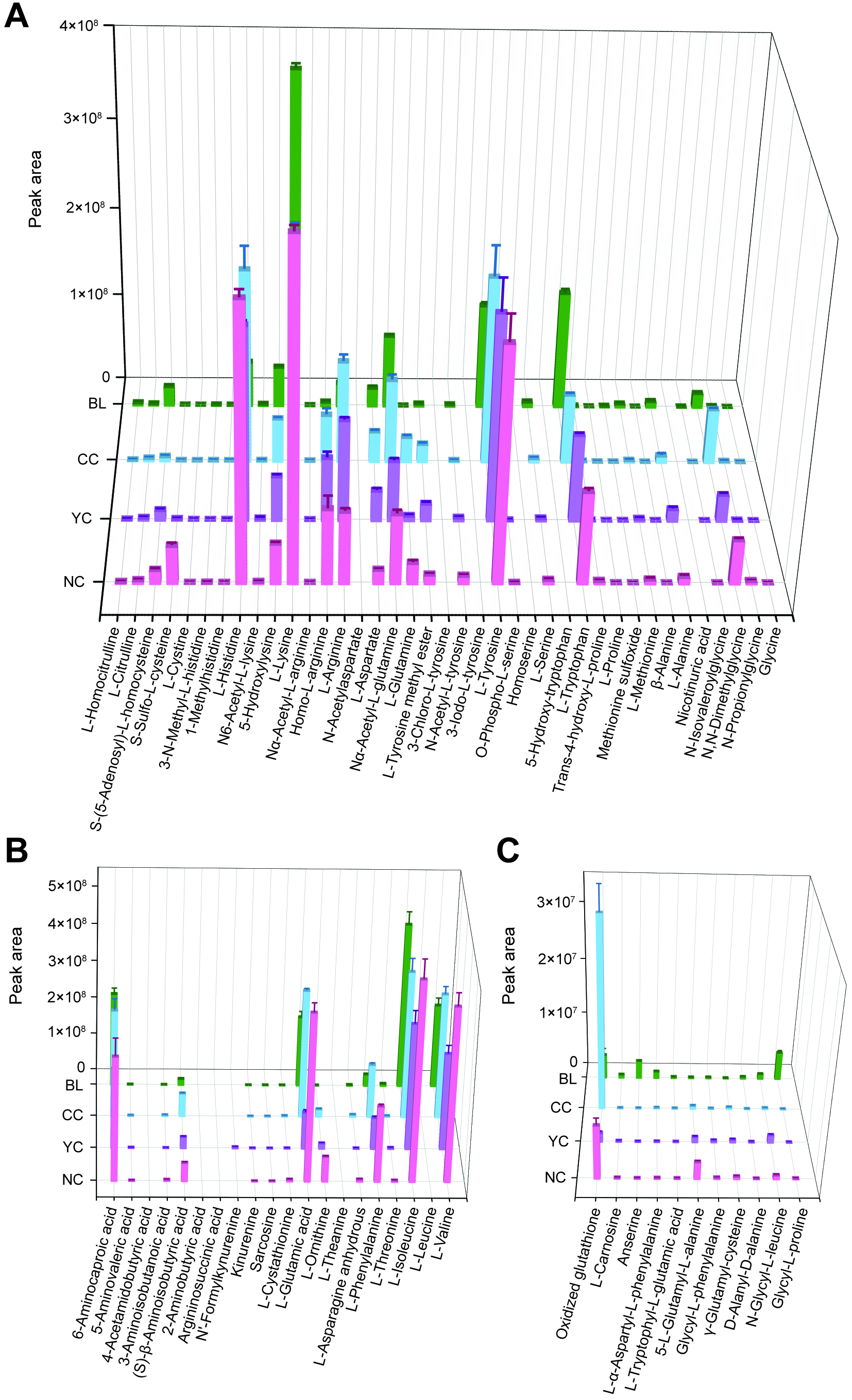

Supplement: Supplementary file 1 [file Image2.TIF]

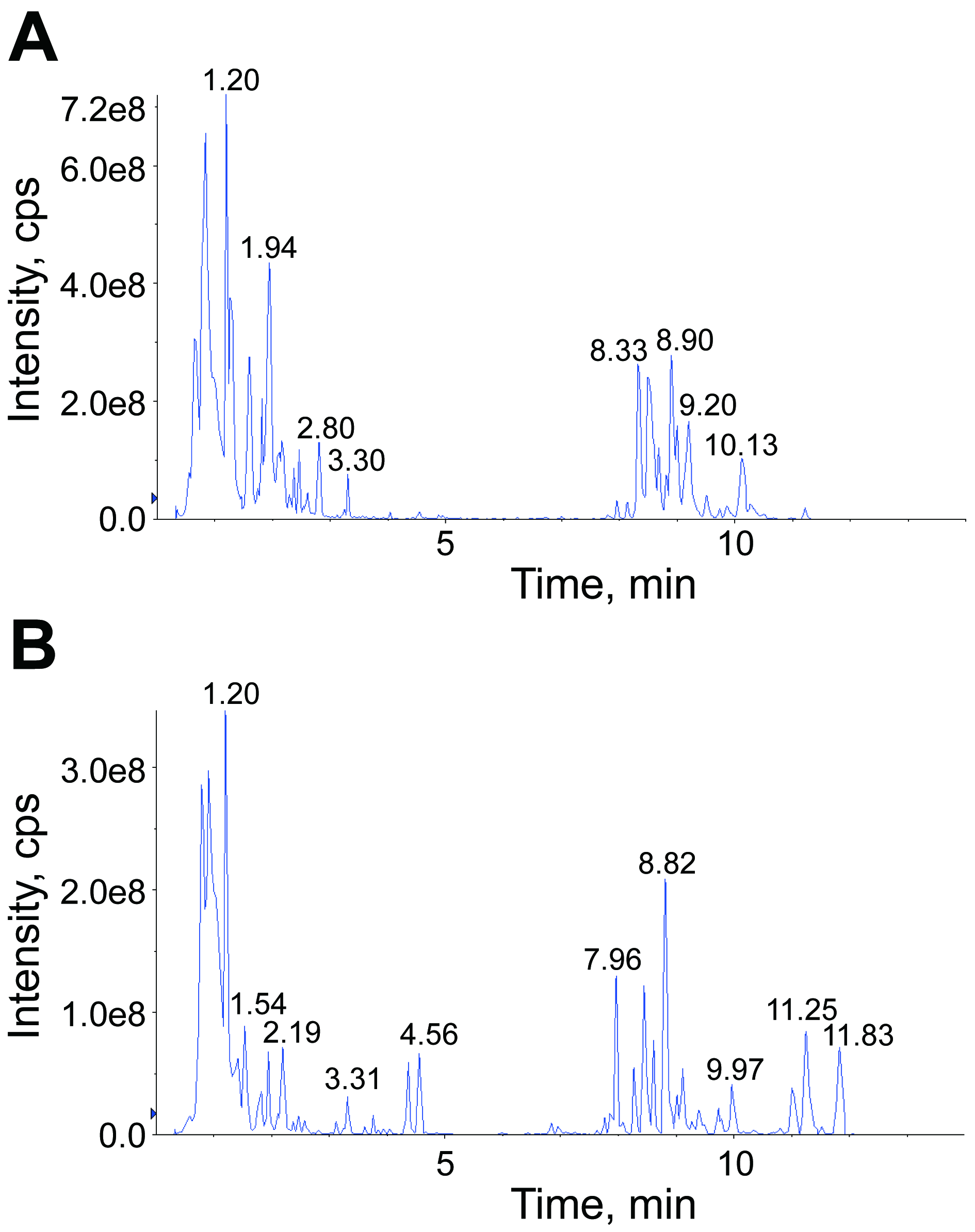

Supplement: Supplementary file 2 [file Image1.TIF]
